# The impact of interleukin-6 receptor inhibitors on risk of diabetes mellitus in patients with giant cell arteritis: a cohort study

**DOI:** 10.1007/s00296-026-06189-y

**Published:** 2026-06-26

**Authors:** Mads E. R. Sørensen, Sille Sophie Lindhardt Petersen, Anne Lund Overgaard, Maria Kristina Stilling-Vinther, Esther Gaarn Lindgreen, Lone Salomonsen, Wilfred Kjær Hanefelt Dinesen, Katja Bailum Knudsen, Kathrine Banke Hansen, Martin Jensen, Kirsten S. Duch, Line Uhrenholt, Bolette Gylden Soussi, Lene Wohlfahrt Dreyer, Salome Kristensen

**Affiliations:** 1 Centre for Rheumatic Research Aalborg, Department of Rheumatology, Aalborg University Hospital, Aalborg, Denmark; 2 Department of Clinical Medicine, Aalborg University, Aalborg, Denmark; 3 Research Data and Biostatistics, Aalborg University Hospital, Aalborg, Denmark; 4 Center for Molecular Prediction of Inflammatory Bowel Disease (PREDICT), Department of Clinical Medicine, Aalborg University Copenhagen, Aalborg, Denmark

**Keywords:** Clinical trial, Giant cell arteritis, Comorbidities, Diabetes mellitus, Cardiovascular disease, Osteoporosis, Interleukin-6 receptor inhibitor, Tocilizumab, Glucocorticoids, Cohort study

## Abstract

To investigate the one-year incidence of diabetes mellitus (DM) among patients with giant cell arteritis (GCA) treated with glucocorticoids and interleukin-6 receptor inhibitor (IL-6Ri) compared to patients treated with glucocorticoid monotherapy. Secondary we investigated the one-year risk of a composite outcome of DM, osteoporosis and cardiovascular events. This cohort study at Aalborg University Hospital, Denmark included patients with GCA from June 2022 to August 2025. Data was collected at diagnosis and at 4, 12, and 24 weeks after glucocorticoid initiation, and annually thereafter. We evaluated the one-year incidence of DM, and thereafter the risk the composite outcome using a Cox regression, comparing patients receiving glucocorticoids in combination with IL-6Ri (exposed) versus glucocorticoid monotherapy (unexposed). The study included 121 patients (70.2% female; mean age 70.5 years): 47 exposed and 74 unexposed. A median of 193 days passed from GCA diagnosis to IL-6Ri initiation in exposed. The one-year cumulative incidence of DM was 3.0% in exposed and 17.5% in unexposed. Exposed had a lower composite risk (Hazard ratio: 0.3, 95% confidence interval: 0.1;1.3). The most frequent AEs among exposed were infections, osteoporosis, cardiovascular events, and DM in exposed and unexposed. Both the one-year cumulative incidence of DM and the composite risk of adverse events were lower in exposed compared with unexposed. These findings suggest that IL-6Ri may offer a safer alternative to glucocorticoid monotherapy in reducing long-term risks. Additionally, the data indicate that IL-6Ri may be introduced too late in the disease course.

**Supplementary Information:**

The online version contains supplementary material available at 10.1007/s00296-026-06189-y.

## Introduction

Giant cell arteritis (GCA) is the most common systemic vasculitis, which causes chronic inflammation of large and medium-sized arteries in individuals aged ≥ 50 years [1]. In Denmark, the annual incidence rate of GCA is among the highest in the world, with approximately 19–25:100.000 persons > 50 years [2] and a female-to-male ratio of 2:1 [3]. Despite their significant toxicity, glucocorticoids remain the primary treatment for GCA, typically administered with a planned taper over 12 months. However, as relapses occur in up to 50% of patients during tapering, many remain in need of glucocorticoid therapy for years [4, 5]. The prolonged duration of treatment carries significant consequences, with up to 86% of patients with GCA experiencing adverse events (AE) associated with glucocorticoid treatment [6]. Among the most common AEs are arterial hypertension, osteoporosis, cardiovascular events, infections, and diabetes mellitus (DM) [6–14]. A Danish study from 2017 found a sevenfold higher dose-dependent risk of DM during the first year of disease compared to matched controls from the general population [14]. A 2017 observational study found that patients with GCA receiving 30 mg/day glucocorticoids, had an almost twofold dose-dependent increased risk of osteoporosis [15]. The one-year cumulative incidence of glucocorticoid-associated cardiovascular events was examined in a 2020 retrospective cohort study, which found a dose-dependent incidence of 8.9% in patients treated with ≥ 25.0 mg/day [16].

To reduce the severe AEs associated with glucocorticoid therapy, studies have evaluated the interleukin-6 receptor inhibitor (IL-6Ri), an anti-cytokine agent, as a glucocorticoid-sparing strategy [17, 18]. This evidence is incorporated in the 2018 European Alliance of Associations for Rheumatology (EULAR) guidelines, which recommend adjunctive IL-6Ri treatment for patients with relapsing disease and those at increased risk of glucocorticoid-induced AEs, such as individuals with pre-existing comorbidities [19]. However, in clinical practice, IL-6Ri treatment is often introduced relatively late in the course of the disease. Furthermore, the impact of IL-6Ri on the occurrence of AEs is not well established, and it is likely that the delayed treatment initiation cause an observed decrease in the development of AEs. Moreover, since glucocorticoids will likely remain a cornerstone in treatment of patients with GCA, it is not feasibly to fully eliminate glucocorticoid-induced AEs. To improve the treatment regimen, it is important to evaluate the impact of glucocorticoid-sparing agents on the risk of AEs and how IL-6Ri affects unselected patients in a real-world clinical setting.

### Objectives

The primary aim of this study was to investigate the one-year incidence of DM in patients with GCA treated with glucocorticoids and IL-6Ri compared to patients treated with glucocorticoid monotherapy. The secondary aim was to investigate whether treatment with the glucocorticoid-sparing agent IL-6Ri reduces the composite risk of DM, osteoporosis, and cardiovascular events.

## Materials and methods

The method and statistical analyses will be reported according to recommendations from the Enhancing the Quality and Transparency Of health Research (EQUATOR) network [20]: the Strengthening the Reporting of Observational Studies in Epidemiology (STROBE) statement [21, 22]. The Strobe checklist can be found as supplementary material.

### Study design and setting

This prospective cohort study was conducted at the Department of Rheumatology at Aalborg University Hospital, Denmark. Enrolment took place at routine visits in the outpatient clinic. By the time of inclusion, all patients received oral and written information and provided oral and written consent. Patients were included from June 2022 to August 2025. Data collection continued until October 2025. The study was approved by the ethics committee of Northern Jutland (N-20220009) and registered in the North Denmark Region internal list of research projects (F2022-015).

### Study population

Patients between the age of 50 and 85 diagnosed with GCA by a rheumatologist were considered eligible in the study. The included patients received treatment in accordance with the National Danish GCA management guideline [23]. Patients were excluded if they had a history of psychiatric or psychological conditions that would affect their ability to participate, if they were unable to provide informed consent, unwilling to comply with the study protocol, or considered unsuitable by the clinician.

### Variables

The primary endpoint was the one-year cumulative incidence of DM, which was defined as a HbA1c ≥ 48 mmol/mol and/or newly prescribed antidiabetic medication and/or an ever blood glucose above 200 mg/DL. Data on fasting blood glucose was not available. The secondary endpoint was time to first event of a composite outcome consisting of DM, osteoporosis, and cardiovascular events. Osteoporosis was defined, according to the Danish Endocrine Society, as a T-score ≤ -2,5 in the vertebral column or the hip or the occurrence of a low-energy fracture in a vertebra, the hip, or the femur [24]. To screen for osteoporosis, a DXA scan was performed at initiation of glucocorticoids (within twelve weeks), and at approximately two years, and four years after first scan. Cardiovascular events were recorded when documented in patients’ hospital records. The cardiovascular events included myocardial infarction, transient cerebral ischemia, cardiac insufficiency/failure, stroke, and cerebral hemorrhage, which is based on previous literature [25].

### Exposure and comparator group

The exposure group consisted of patients diagnosed with GCA treated with IL-6Ri in combination with glucocorticoids (denoted exposed in the rest of this paper). The comparator group consisted of patients diagnosed with GCA treated with glucocorticoids monotherapy (denoted unexposed in the rest of this paper). Exposed were considered at risk from the first day of receiving IL-6Ri (index date), until the end of follow-up, they withdrew their consent, were discharged from the outpatient clinic, or died, whichever came first. Unexposed were considered at risk from the first day of receiving glucocorticoids as treatment for GCA (index date), until the end of follow-up, they withdrew their consent, received IL-6Ri, were discharged from the outpatient clinic, or died, whichever came first. For exposed, no restrictions on time between diagnosis and treatment with IL-6Ri were set; however, we conducted sensitivity analyses in which exposed had to start treatment with IL-6Ri within three months of GCA diagnosis.

### Data sources

Data were obtained from patient interviews and hospital records at the time of diagnosis, at 4, 12, and 24 weeks following glucocorticoid initiation, and annual thereafter. All study visits coincided with patients’ routine outpatient clinic appointments. Patients could be included at any time during the disease course. Additional data was collected and entered as an extra visit in case of a relapse, treatment modification, or development of an AE. A relapse was defined as reappearance of GCA symptoms, elevated acute phase reactants (not due to another apparent cause e.g. infection), or both, resulting in treatment intensification. Infections were registered when they required hospitalization or treatment with antibiotics, antifungals, or antivirals.

At inclusion, the following data were collected: demographics; pre-existing comorbidities; time of diagnosis; glucocorticoid initiation; symptom debut; initial glucocorticoid dose; symptoms; objective findings; blood samples; diagnostic imaging; and fulfilment of GCA classification criteria. At inclusion and annual visits, the patients were asked about their smoking status, weight, and height.

Subsequently, at each visit, the following data were collected: current and cumulative glucocorticoid dose; number of relapses; treatment modification; disease-related symptoms; treatment-related AEs developed since the last visit; initiation of treatment for newly developed comorbidities; use of glucocorticoid-sparing agents; and blood samples. At each visit and when IL-6Ri was initiated, the cumulative glucocorticoid dose was calculated based on tapering plans described in the patients’ hospital records. If the tapering plan was unavailable or the patient had been treated by their general practitioner before enrolment, the cumulative glucocorticoid dose was estimated using the Danish national treatment guideline [23]. Glucocorticoids prescribed for other indications were included in the cumulative glucocorticoid dose.

All data were entered into the electronic case report form in REDCap.

### Statistical analysis

When descriptive statistics were performed, the Shapiro-Wilk test was used to test for normality. If normally distributed, continuous variables were presented as mean and standard deviation (SD), whereas non-normally distributed variables were presented with median and interquartile range (IQR). Categorical variables were presented as numbers and percentages. A p-value of < 0.05 indicated statistical significance. The one-year cumulative incidence was calculated for the development of DM from the index date in both cases and unexposed. In this study, we also used Cox regression with time since index as underlying time scale, to calculate one-year hazard ratio (HR) with a 95% confidence interval (Cl) for development of a composite outcome of DM, osteoporosis, and cardiovascular events in exposed compared to unexposed. We accounted for patients contributing to both exposure groups by applying robust standard errors. No power calculation was performed due to the observational study design, which included all eligible patients during the study period.

Patients with pre-existing DM, osteoporosis, or a cardiovascular event were excluded from the statistical analysis. The combination of DM, osteoporosis, and cardiovascular events in the composite outcome was chosen as AEs of interest, since these are some of the most frequent glucocorticoid-associated AEs. The proportional hazards assumption was evaluated both graphically by examining the survival curves and statistically using Schoenfeld residuals. If more than 10% of the data were missing, this was denoted in the tables and excluded in the statistical analysis. Data were analyzed using RStudio version 4.5.1.

## Results

### Baseline demographics and disease characteristics

**Fig. 1:**
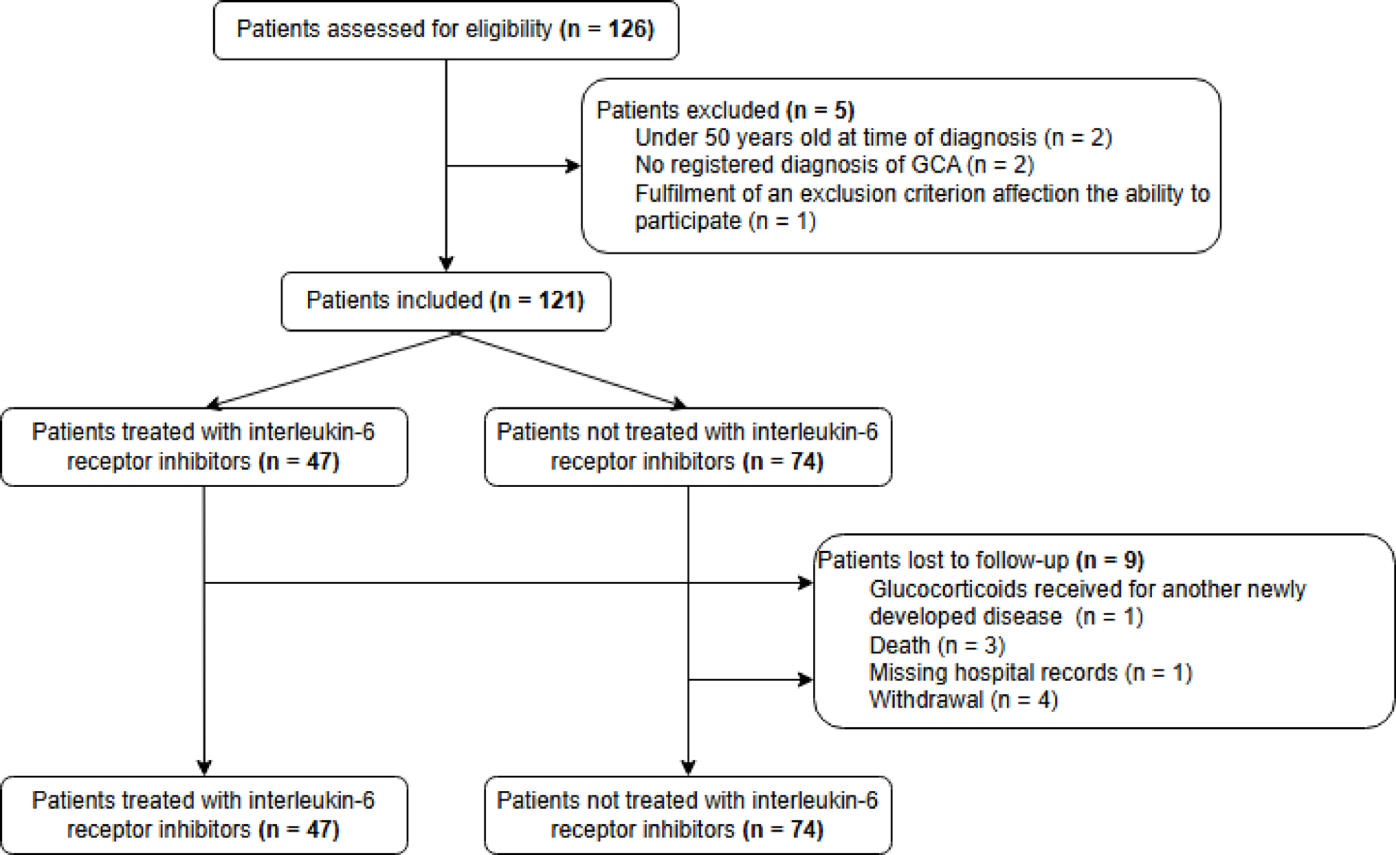
Flowchart of the patients included in the study

A total of 126 patients were considered eligible. Five patients were excluded, as shown in Fig. 1, resulting in 121 patients with GCA being included. Table 1 presents baseline demographics and disease characteristics for all included patients, no matter if they were excluded in the statistical analysis. During the study period, 47 (38.8%) patients were treated with IL-6Ri after a median of 193 (IQR: 44;459) days.

**Table 1:** Baseline demographics and disease characteristics of patients with giant cell arteritis treated with interleukin-6-receptor inhibitors in combination with glucocorticoids (exposed) or glucocorticoid monotherapy (unexposed)

	Exposed (*n* = 47)	Unexposed (*n* = 74)
Female, *n* (%)	36 (76.6)	49 (66.2)
Age in years, mean (SD)	69.6 (8.7)	72.1 (7.3)
Smoking status, n (%)		
Current smoker	4 (16.6)	5 (11.36)
Former smoker	8 (37.5)	21 (52.3)
Never-smoker	9 (48.8)	15 (36.4)
Missing	26 (55.3)	33 (44.6)
BMI, median (IQR)	25.4 (23.2; 29.2)	26.2 (24.1; 28.9)
Missing, n (%)	32 (68.1)	35 (47.3)
Dose of glucocorticoid mg/day at index date, median (IQR)	25.0 (15.0;40.0)	60.0 (40.0; 60.0)
Days from start of glucocorticoids to start of IL-6Ri, days,median (IQR)	193 (44.0; 459.0)	
Diagnosis, n (%)		
GCA, total	25 (53.2)	40 (54.1)
* c-GCA*	16 (34.0)	26 (35.1)
*LV-GCA*	4 (8.5)	9 (12.2)
*LV-GCA + c-GCA*	5 (10.6)	5 (6.8)
GCA + PMR, total	22 (46.8)	34 (45.9)
*c-GCA + PMR*	13 (27.7)	24 (32.4)
*LV-GCA + PMR*	6 (12.8)	6 (8.1)
*LV-GCA + c-GCA + PMR*	3 (6.4)	4 (5.4)
Comorbidities, n (%)	44 (93.6)	50 (67.6)
Arterial hypertension	26 (55.3)	31 (41.9)
Hyperlipidemia	10 (21.3)	16 (21.6)
Diabetes mellitus	12 (25.5)	2 (2.7)
Osteoporosis	20 (42.6)	8 (10.8)
Solid malignancy	3 (6.4)	7 (9.5)
Neuropsychiatric disease	5 (10.6)	9 (12.2)
Cardiovascular event	8 (17.0)	17 (23.0)
Clinical symptoms and findings at diagnosis, n (%)		
Morning stiffness in shoulder and neck	14 (31.1)	33 (44.6)
Sudden visual loss	5 (11.1)	10 (13.5)
Jaw or tongue claudication	20 (44.4)	38 (51.4)
New temporal headache	36 (80.0)	53 (71.6)
Scalp tenderness	18 (40.0)	31 (41.9)
Reduced pulse at the temporal artery	4 (8.9)	6 (8.1)
CRP ≥ 10 mg/L or SR ≥ 50 mm/hour	44 (97.8)	71 (95.9)

n: number, SD: standard deviation, BMI: body mass index, mg: milligram, IQR: interquartile range, IL-6Ri: interleukin-6-receptor inhibitor, GCA: giant cell arteritis, c-GCA: cranial gca, LV-GCA: large-vessel GCA, PMR: polymyalgia rheumatica, CRP: c-reactive protein, L: liter, SR: sedimentation rate, mm: millimeter

Among exposed, the most prevalent comorbidities were arterial hypertension (55.3%), osteoporosis (42.6%), hyperlipidemia (21.3%), and DM (25.5%), compared with 41.9%, 10.8%, 21.6%, and 2.7%, respectively, in unexposed. Median initial glucocorticoid doses at the index date were lower in exposed than unexposed (25.0 mg, IQR: 15.0–40.0 vs. 60.0 mg, IQR: 40.0–60.0). Baseline demographics for the sensitivity cohort are provided in Supplementary Table S1.

### Adverse events

Table 2 summarizes the AEs observed during the study period. Among exposed, one patient developed DM, corresponding to a one-year cumulative incidence of 3.0%. In comparison, 11 unexposed developed DM, yielding a one-year cumulative incidence of 17.5% following glucocorticoid initiation. In both groups, all cases of DM occurred within six months of the index date.

**Table 2:** Adverse events developed during the study period in patients with giant cell arteritis treated with interleukin-6-receptor inhibitors in combination with glucocorticoids (exposed) or glucocorticoid monotherapy (unexposed)

	Exposed (*n* = 47)	Unexposed (*n* = 74)	*P*-value
Adverse events, *n* (%)	27 / 47 (57.5)	40 / 74 (54.1)	0.05
Arterial hypertension*	1 / 21 (4.8)	7 / 43 (16.3)	0.02
Cardiovascular events	5 / 47 (10.6)	9 / 74 (12.2)	0.65
Diabetes mellitus*	1 / 35 (2.9)	11 / 72 (15.3)	< 0.001
Aggravation of pre-existing diabetes mellitus	5 / 12 (41.6)	1 / 2 (50.0)	0.24
Osteoporosis*	3 / 27 (11.1)	9 / 66 (13.6)	< 0.001
Neuropsychiatric disease*	0 / 42 (0.0)	4 / 65 (6.2)	0.46
Weight gain (> 3 kg)	3 / 47 (7.5)	5 / 74 (6.8)	0.26
Infections*	20/ 47 (42.6)	23 / 74 (31.1)	0.01
Risk of developing adverse events (diabetes mellitus, osteoporosis, or a cardiovascular event), HR (Cl)*	0.3 (0.1;1.3)	1.0 (ref)	
Development of diabetes mellitus, n (%)*			
4 weeks	0/34 (0.0)	6 / 72 (8.3)	
3 months	0/34 (0.0)	4 / 66 (6.1)	
6 months	1/34 (2.9)	1 / 65 (1.5)	
12 months	0/34 (0.0)	0 / 65 (0.0)	
24 months	0/34 (0.0)	0 / 65 (0.0)	
One-year cumulative incidence of Diabetes mellitus*	3.0	17.5	

*Patients with a prior diagnosis were excluded

n: number, kg: kilograms, HR: hazard ratio, Cl: confidence interval

Apart from DM, the most frequent AEs among exposed were infections (44.7%), osteoporosis (10.7%), cardiovascular events (8.5%), and arterial hypertension (4.8%). For unexposed, the corresponding incidences were 31.1% for infections, 13.6% for osteoporosis, 12.2% for cardiovascular events, and 16.3% for arterial hypertension. As seen in Table 2, for arterial hypertension (*P* = 0.02), DM (*P* < 0.001), osteoporosis (*P* < 0.001) and infections (*P* = 0.01) the difference between groups were all significant. No included patients had any osteoporotic fractures. When performing the sensitivity analysis, in which exposed had to be treated with IL-6Ri within three months of GCA diagnosis, the percentage of comorbidities developed remained the same (Supplementary Table S2).

As shown in Supplementary Table S3, a proportion of exposed developed comorbidities in the interval between glucocorticoid initiation and the start of IL-6Ri treatment (index date). During this period, the incidence were as follows: DM 7.9%; infections 31.9%; osteoporosis 24.3%; arterial hypertension 8.7%; and cardiovascular events were 6.4%.

Although treatment with IL-6Ri appeared to be associated with a reduced risk of the composite outcome of DM, osteoporosis, or cardiovascular events (HR: 0.3, 95% confidence interval: 0.1;1.3), the association did not reach statistical significance.

In Table 3, the cumulative glucocorticoid doses and relapse rates for exposed and unexposed can be seen.

**Table 3:** Cumulative glucocorticoid doses and relapse rates in patients with giant cell arteritis treated with interleukin-6-receptor inhibitors in combination with glucocorticoids (exposed) or glucocorticoid monotherapy (unexposed)

	Exposed (*n* = 47)	Unexposed (*n* = 74)
Cumulative glucocorticoid dose, mg, median (IQR), Missing, *n* (%)		
Before index date	4800.0 (2105;8813)	0.0
3 months after index date	1345 (840;1780), 2 (4.3)	1790 (1350;2227), 8 (10.8)
6 months after index date	2063 (1537;2348), 2 (4.3)	3510 (2693;4307), 11 (14.9)
12 months after index date	2634 (1920;3648), 3 (6.4)	4705 (3472;5455), 4 (5.4)
24 months after index date	3388 (2042;5210), 9 (19.1)	5809 (5015;7093), 22 (30.0)
Patients with at least one relapse, n (%)	21 (44.7)	47 (63.5)

n: number, mg: milligram, IQR: interquartile range

## Discussion

In this study, 3.0% of exposed developed DM within the first six months after the index date, compared with 17.5% of unexposed. These findings suggest that IL-6Ri therapy may reduce the risk of DM in patients with GCA treated with glucocorticoids. To our knowledge, no previous studies have specifically examined the incidence of glucocorticoid-induced DM in patients receiving combined glucocorticoid and IL-6Ri therapy compared with glucocorticoid monotherapy. However, a French study published in 2021 reported a 26% prevalence of DM prior to initiation of IL-6Ri and 7% after initiation among 43 patients with GCA treated with IL-6Ri, which is similar to results in this study [26]. Collectively, the findings from the present study and the French cohort support a potential protective effect of IL-6Ri on the development of glucocorticoid-associated DM in patients with GCA.

The secondary objective of this study was to assess whether IL-6Ri treatment reduces the composite risk of DM, osteoporosis, and cardiovascular events. In our cohort, exposed exhibited a lower, though not statistically significant, composite risk of DM, osteoporosis, or cardiovascular events compared with unexposed. Similarly, a Swiss phase II, randomized, double-blind, placebo-controlled trial published in 2016 reported fewer cardiovascular events and osteoporotic fractures in patients treated with IL-6Ri plus glucocorticoids (*n* = 20) compared with those receiving glucocorticoids plus placebo (*n* = 10) [27].

In this study, exposed received lower glucocorticoid doses than unexposed during follow-up. At the index date, exposed were receiving less than half the glucocorticoid dose administered to unexposed (Table 1), likely reflecting that the index date for exposed patients was defined at initiation of IL-6Ri, which on average occurred 193 days after the index date of unexposed patients (Table 1). Several studies have demonstrated a dose-dependent association between GC exposure and development of DM and osteoporosis in patients with GCA [14, 15]. Importantly, this difference was not limited to baseline dosing: as shown in Table 3, exposed patients also accumulated approximately half the cumulative glucocorticoids dose during the 24 months following the index date compared with unexposed. This difference in glucocorticoid exposure is likely to have had a significant impact on the risk of adverse events.

Given the well-established dose-dependent relationship, the risk of adverse events is greatest in the early phase of treatment, when glucocorticoid doses are highest. Correspondingly, in most exposed, the period of highest AE-risk occurred prior to initiation of IL-6Ri therapy. This is supported by the observation that all DM events in the control group occurred within the first six months, and three out of four DM events occurred before IL-6Ri initiation in the exposed group (Supplementary Table S3). Therefore, many patients predisposed to developing, e.g., DM may already have done so before initiating IL-6Ri. This could lead to an overestimation of the protective effect of IL-6Ri, as the relatively low number of incident comorbidities observed after treatment initiation may partly reflect a high pre-existing burden. Also, as the index date for exposed is the date of IL-6Ri-initiation, exposed have accumulated large doses of glucocorticoids before initiation, and as seen in Table 3, approximately 1300 mg more during the same period compared to unexposed. However, afterwards cases accumulated lower doses, which complicates interpretation of any causal inference of glucocorticoids.

According to EULAR recommendations, indications for IL-6Ri therapy include relapsing or refractory disease, as well as patients with established or high risk of glucocorticoid-related comorbidities. As exposed in this study started IL-6Ri treatment a median of 193 days after diagnosis with GCA, and 53.2% developed one or more AEs before initiation of IL-6Ri, which heavily introduces substantial confounding by indication. On one hand, such confounding might be expected to underestimate the protective effect of IL-6Ri, as patients selected for treatment represent a higher-risk population. On the other hand, because many exposed initiated IL-6Ri after the development of comorbidities, these events are classified as occurring prior to IL-6Ri initiation, which may overestimate the protective effect. However, the above mentioned bias does complicate the interpretation of any protective effect of IL-6Ri.

These considerations collectively suggest that IL-6Ri therapy may often be introduced relatively late in the disease course, after significant glucocorticoid exposure and the development of comorbidities have already occurred.

Interestingly, when assessing the risk of AEs in general, this study found that 53.2% of exposed developed one or more AEs before initiation of IL-6Ri (Supplementary Table S3) and 57.5% after (Table 2). An American observational study from 2021 that investigated the safety of IL-6Ri in patients with GCA found an incidence of 48.3% before and 53.3% after IL-6Ri initiation [28]. Similarly, the former-mentioned French study found that 84% developed AEs before, and 95% after, initiation of IL-6Ri [26]. Even though the results are similar to those of the previous studies, there are obvious differences in the AEs investigated. For instance, the French study found neutropenia and corticotropin deficiency among their most frequent AEs; these were not investigated in this study. Furthermore, both aforementioned studies included osteoporotic fractures, which may occur in patients with a pre-existing diagnosis of osteoporosis, whereas this study included only newly developed osteoporosis. Both the American and the French study investigated the most frequent AEs associated with both IL-6Ri and glucocorticoid treatment, whereas this study primarily investigated known AEs associated with glucocorticoid treatment [26, 28]. The American study reported that 88.1% of the AEs developed before IL-6Ri initiation were attributed to glucocorticoids, compared to 44.4% of AEs occurring after IL-6Ri initiation [26]. These findings support the results of the present study in which fewer glucocorticoid-related AEs were observed in patients treated with IL-6Ri. The incidence of AEs in patients treated with IL-6Ri has also been reported in the GiACTA study, a randomized, double-blind, placebo-controlled, phase III trial, that compared patients receiving glucocorticoids plus IL-6Ri with patients receiving glucocorticoids plus placebo [17]. The study found that 96% of the patients treated with IL-6Ri every two weeks experienced AEs, compared to 92% in patients receiving glucocorticoids plus placebo for 52 weeks. When assessing severe AEs, the GiACTA study found an incidence of 14% in the IL-6i-group compared with 25% in the glucocorticoids plus placebo-group [17]. However, apart from the severe AEs, the overall risk of AEs was not reduced.

This study has several limitations. First, the study population was small, a point that was particularly evident in the statistical subgroup analyses. Before performing the Cox proportional regression analyses, all patients with pre-existing DM, osteoporosis, or a cardiovascular event were excluded, resulting in only 18 exposed and 68 unexposed being included in the analysis, which in combination with few events, makes it hard to draw any conclusions. Because the index date for exposed patients is the date of IL-6Ri initiation, which occurs a median of 193 days after diagnosis of GCA, patients must remain alive and free of events up to treatment initiation. This artificially reduces the observed AE rates. Consequently, some patients with the greatest burden of comorbidities were excluded from the analysis, potentially underestimating the total number of events in the study population. As seen in Table 1, there is an imbalance in comorbidities between exposed and unexposed, which further complicates any casual conclusions. Moreover, the model did not account for the possibility of multiple events. Because only a few patients developed each AE, it was not possible to perform individual regression analyses. Additionally, due to the time imbalance in the two groups, immortal time bias is induced, which also complicates the interpretation of our findings, since the index date of exposed in this study was the day of IL-6Ri treatment. If death or other events occurred before IL-6Ri initiation, these patients would be censored from the Cox proportional hazards regression analysis, potentially overestimating the effect of IL-6Ri.

A strength of this study was the ability to include the majority of patients with GCA from Northern Jutland, as it is recommended that all patients with symptoms of GCA be referred to a specialized hospital department. Lastly, this study assessed patients in a real-world clinical setting with few inclusion and exclusion criteria, making the results more transferable to clinical practice.

In conclusion, this study found that the one-year cumulative incidence of DM was 3.0% among exposed and 17.5% among unexposed. Additionally, a tendency towards a reduced composite risk of DM, osteoporosis, or cardiovascular events in patients treated with IL-6Ri was observed, although not statistically significant. Thus, treatment with IL-6Ri may reduce the risk of glucocorticoid-associated AEs. Given the regulatory and clinical constraints surrounding IL-6Ri initiation, this study represents a challenging clinical dilemma. The results of this study support early identification of high-risk individuals and more timely initiation of glucocorticoid-sparing strategies. However, due to the small sample size and low number of events in this study, further studies investigating benefits and safety of IL-6Ri in real-world clinical settings is needed to improve the treatment regimen of GCA.

## Supplementary Information

Below is the link to the electronic supplementary material.

Supplementary Material 1

Supplementary Material 2

## Declarations

### Generative AI tools

In this study, generative AI tools (ChatGPT) were used to improve the grammar and clarify the language, as well as optimize and problem-solve the code in RStudio. No sections were written by AI tools, and no analyses were carried out by AI. All output from the AI tools was critically evaluated by the authors before applying it in the study. No part of this report is copied, published elsewhere, or generated by AI.
