# Supplementary material for: The impact of interleukin-6 receptor inhibitors on risk of diabetes mellitus in patients with giant cell arteritis: a cohort study
Source: Rheumatol Int. 2026 Jun 26;46(7):170. doi: 10.1007/s00296-026-06189-y (PMC13309367; doi:10.1007/s00296-026-06189-y)
Supplement: Supplementary file 2 — Supplementary Material 2 [file 296_2026_6189_MOESM2_ESM.docx]

Supplementary material

| *Supplementary Table S1: Baseline demographics and disease characteristics of patients with giant cell arteritis treated with interleukin-6-receptor inhibitors within 3 months of diagnosis (exposed) or treated with glucocorticoids monotherapy (unexposed)* | | |
| --- | --- | --- |
|  | Exposed (n=19) | Unexposed (n=102) |
| Female, n (%) | 14 (73.7) | 71 (69.6) |
| Age in years, mean (SD) | 71.5 (6.9) | 70.5 (8.2) |
| Smoking status, n (%)  Current smoker  Former smoker  Never-smoker  Missing | 1 (5.3)  5 (26.3)  7 (36.8)  6 (31.6) | 8 (7.8)  24 (23.5)  17 (16.7)  53 (52.0) |
| BMI, median (IQR)  Missing, n (%) | 25.4 (21.5;27.5)  8 (42.1) | 25.9 (22.6;28.6)  15 (14.7) |
| Dose of glucocorticoid mg/day at index date, median (IQR) | 40.0 (20.0;50.0) | 50.0 (40.0;60.0) |
| Days from start of glucocorticoids to start of IL-6Ri, days, median (IQR) | 45 (28.0;79.0) |  |
| Comorbidities, n (%)  Arterial hypertension  Hyperlipidemia  Diabetes mellitus  Osteoporosis  Solid malignancy  Neuropsychiatric disease  Cardiovascular event | 19 (100.0)  13 (68.4)  6 (31.6)  9 (47.4)  9 (47.4)  1 (5.3)  2 (10.5)  5 (26.3) | 67 (65.7)  42 (41.2)  20 (19.6)  4 (3.9)  13 (12.7)  9 (8.8)  11 (10.8)  18 (17.6) |
| *n: number, SD: standard deviation, BMI: body mass index, mg: milligram, IQR: interquartile range, IL-6Ri:* interleukin-6-receptor inhibitor | | |

| *Supplementary Table S2: Adverse events developed during the study period in patients with giant cell arteritis treated with interleukin-6-receptor inhibitors within 3 months of diagnosis (exposed) or treated with glucocorticoids monotherapy (unexposed)* | | |
| --- | --- | --- |
|  | Exposed (n=19) | Unexposed (n=102) |
| Adverse events, n (%)  Arterial hypertension*  Cardiovascular events  Diabetes mellitus*  Aggravation of pre-existing diabetes mellitus  Osteoporosis*  Neuropsychiatric disease*  Weight gain (>3 kg)  Infections | 14 / 19 (73.7)  1 / 6 (16.6)  2 / 19 (10.5)  0 / 10 (0.0)  1 / 9 (11.1)  2 / 10 (20.0)  3 / 17 (17.6)  3 / 19 (15.8)  10/ 19 (52.6) | 59 / 102 (57.8)  10 / 60 (16.6)  12 / 102 (11.8)  12 / 98 (12.2)  3 / 4 (75.0)  16 / 89 (18.0)  4 / 65 (6.2)  5 / 91 (5.5)  37 / 102 (36.3) |
| *n: number, kg: kilograms*  **Patients with a prior diagnosis were excluded.* | | |

| *Supplementary Table S3: Adverse events developed between start of glucocorticoid treatment and index date in exposed* | |
| --- | --- |
|  | Exposed (n=47) |
| Adverse events, n (%)  Arterial hypertension*  Cardiovascular events  Diabetes mellitus*  Aggravation of pre-existing diabetes mellitus  Osteoporosis*  Neuropsychiatric disease*  Weight gain (>3 kg)  Infections | 25 / 47 (53.2)  2 /23 (8.7)  3 /47 (6.4)  3 /38 (7.9)  4 /9 (55.6)  10 /37 (24.3)  2 /47 (4.5)  7 /47 (14.9)  15 /47 (31.9) |
| *n: number, kg: kilograms*  **Patients with a prior diagnosis were excluded.* | |
